# Beauty’s Tyranny

**Published:** 1882-05

**Authors:** 


### BEAUTY'S TYRANNY.

Beauty is pleasant to gaze upon at all times, but it is particularly so in the person of a woman. Not only is it pleasant to gaze upon, but it possesses a mighty power of fascination. Men who will remain unconvinced by hours of lengthy, logical argument, will hopelessly succumb to its influence and do what they know to be wrong for its sake. To win a smile from it persons have ere now plunged themselves into abysses of sin and folly, and there is no reason to suppose that the men of the future will act differently than the men of the past have acted. It has drawn money from the pockets of misers; it has called smiles to faces which have become wrinkled with much scowling; and it has made the greedy act as if they were benevolent. Beggars are wide enough awake to know that they have a better chance of wheedling something from the purse of the skinflint when he is leaning on the arm of beauty than at any other time, and they act accordingly. People do not like to appear mean or debased when they are in the presence of beauty; they wish to appear even better than they are at such a time, so that they may be considered fit to stand on its exalted platform; and hence it is that they act in the manner indicated. Now, beauty is one of the most valuable gifts of God; it is good for men to attempt to raise themselves at any time; and it is well that people should perform generous actions now and then, but it is unreasonable for them to do so solely for the purpose of propitiating beauty, for, as a matter of fact, beautiful women are not as a class, in spite of the verdict of a host of novelists to the contrary, any better than their plainer sisters. Indeed, it is questionable whether, as a whole, they are so good as the latter. Necessarily, the woman who possesses an uncommon share of good looks is vainer than the woman who is the owner of but a paucity of the same; the flattery to which she is subjected naturally makes her so. She would be something more than human if she could be the daily recipient of spoken and implied compliments and remain as modest as she would were she compelled to exist in a sphere in which there were no flatterers and no mirrors. She would be a wonder, indeed, if she could see crowds of admirers flocking to her side, while less gifted females stand neglected, and yet refrain from imagining that she is altogether a very superior sort of mortal. She would be beyond all praise if she could hear the merits of others systematically detracted from in order that hers may appear the greater, and yet not believe that in many respects she is the quintessence of excellence. Then admiration prompts people to perform a thousand little offices for her which many of her fellows are left to perform for themselves, and which, it may be remarked, they are all the better for performing for themselves. Nor is this all. Though a great deal of the flattery which is paid to beauty is sincere, a great deal is quite the reverse and absurdly hyperbolical. There are men who are ready to descend to any depth of hypocrisy in order to deceive a woman ; there are men who take delight in dosing her with humbug, and who—perhaps justly—imagine that they cannot reasonably hope to secure her favor by any other means. They do not hesitate to tell her falsehoods by the score ; they do not hesitate to indulge in the most drivelling inanity in order to accommodate themselves to her. Such is sometimes the influence of beauty !

It is unfortunately true that beneath a mask of beauty there often lies much that is hideous. This is the case with many pretty women. Their lovely faces and their sylph-like forms are their only merits, if merits they can be called. Shallow of intellect and unfeeling of heart, they have been utterly corrupted by the course of flattery through which they have passed. Of what may be called the higher life they know little and care less. Their one aim is to extort homage from those with whom they are brought in contact. So long as they are successfully doing this they appear to imagine that they are justified in administering snubs right and left, and in holding up to ridicule those who awkwardly attempt to pay homage to them. They take a delight in making their neighbors look small. Obtain an introduction to them, and they will probably declare to your friends that you are a lout. Nor is this all. Unless you are content to bespatter them with praise, unless you are willing to show how deeply impressed you are with their perfections, they will treat you in a very curt manner ; in a manner, in fact, which, if it were adopted by ordinary creatures, would be considered downright rude. They must have, if they are to be satisfied, the best of everything ; and when they cannot get the same, it is discovered that under their beautiful cheeks there lurk tongues of peculiar sharpness. In many cases they cannot love like other women. If one of them has a lover, she feels bound to be continually showing him that she is amazingly condescending to have anything to do with him, and that, unless he acts very humbly and discreetly, she will decline to continue her connection with him. More frequently, however, a woman of the sort in question has no lover in particular, but a great many in general, whom she does not think are nearly good enough for her, and whom she cuts and wounds so unmercifully that, notwithstanding her charms, they desert her one by one, and she at last discovers, to her amazement, that her beauty has faded, and that there is no one, who is worth anything, ready to marry her. Then begins her humiliation. Time goes on, and her charms undergo a further decadence, and at last even her deft manipulation of cosmetics is unable to cope with the ravages which have been made in her beauty. Still, feeling that her beauty is her only possession, she patches herself up, often to such an extent that she becomes positively disfigured. Perhaps she imagines that people do not see through her art. For the sake of her peace of mind it is to be hoped so. But the fact remains that few persons have any compassion for a worn-out beauty, while the great majority take an unholy delight in exposing the artifices which she adopts in order to still secure to herself admiration and flattery. Those over whom the worn-out beauty—(who, in spite of her beauty, is perhaps married to a man she does not love, or else is an old maid)—once triumphed, experience intense delight in expatiating upon the melancholy circumstance that she is decaying before her time ; that she has become as sharp in temper as she is in visage ; and that she is growing a silly, vain and ill-looking creature. Nor is it surprising that spent beauty should be thus treated, for beauty of form and face is but a gift, for which the recipient is not responsible. On the other hand, beauty of mind is an acquirement—often a long and laborious acquirement. Still, though never so potential as beauty of form and face, it is more enduring ; and thus it happens that women who as girls attract little notice outside small circles, as the chariot of time rolls on become queens to whom crowds of subjects pay loving homage. Cannot beautiful women draw a lesson from this ?

---

M. Milne Edwards, in commenting on the results of the deep sea dredgings in the Caribbean sea and the Gulf of Mexico, concludes that a comparison of the abysmal and the littoral animals seems to lay before us two distinct faunæ belonging to different epochs and climates. The animals of the shore deposits belong to higher types. Those of the great depths have a more ancient character, some of them presenting affinities with the fossils of the secondary epoch, and others recalling the larval state of certain recent species.

